# Initial In-Brace Correction: Can the Evaluation of Cobb Angle Be the Only Parameter Predictive of the Outcome of Brace Treatment in Patients with Adolescent Idiopathic Scoliosis?

**DOI:** 10.3390/children9030338

**Published:** 2022-03-02

**Authors:** Angelo Gabriele Aulisa, Marco Galli, Marco Giordano, Francesco Falciglia, Silvia Careri, Renato Maria Toniolo

**Affiliations:** 1 U.O.C. of Orthopedics and Traumatology, Bambino Gesù Children’s Hospital, IRCCS, Piazza Sant’Onofrio 4, 00165 Rome, Italy; agabriele.aulisa@opbg.net (A.G.A.); marco.giordano@opbg.net (M.G.); francesco.falciglia@opbg.net (F.F.); renatomaria.toniolo@opbg.net (R.M.T.); 2 Department of Orthopedic Surgery, University Hospital “Agostino Gemelli”, Catholic University of the Sacred Heart School of Medicine, 00168 Rome, Italy; dottorgalli@yahoo.it

**Keywords:** adolescent idiopathic scoliosis, in brace correction, Risser, brace treatment

## Abstract

Background: Patients with a better initial in-brace correction could show a higher probability of a successful outcome. However, no one has investigated whether parameters can affect the outcomes. The aim of this retrospective study was to evaluate if the initial correction rate (ICR) could be predictive of the bracing outcome and to determine the role of some mechanical and biological parameters in ICR. Methods: The study population comprised 449 patients who met the inclusion criteria. Curve correction > 10° Cobb defines brace treatment success. Success and failure groups were compared in terms of the Risser sign, initial Cobb angle, initial Perdriolle value and ICR. Results: ICR significantly correlates with initial Perdriolle. The success group had a significantly lower value of Pedriolle and initial Cobb angles, Risser sign and ICR than the failure group. The ICR and lower Risser were significantly associated with the brace treatment outcome. This seems particularly suitable for positivity prediction (Predicting value VP+: 87%). Conclusions: This study confirms that immediate in-brace correction can foretell the brace treatment outcome. Patients with a low Risser sign and a high rate of in-brace correction showed a bracing success of 87%. A correlation between rotation and in-brace correction confirms that rotation is among the parameters that influence the deformed spine reaction to corrective actions the most.

## 1. Introduction

The recent literature, with a large number of studies on brace treatment in adolescent idiopathic scoliosis (AIS), has finally highlighted the effectiveness of braces in AIS treatment and put an end to a long discussion on its usefulness. In fact, it can prevent spine deformity progression [1,2,3,4] and, in selected cases, it can produce a partial improvement of the curve [5,6]. Moreover, bracing is effective for long-term results [7,8]: one of author’s studies has already demonstrated a slight loss of correction 15 years after bracing [9].

Knowing the effectiveness of conservative treatment with braces, we need to learn more about the factors that influence the evolutionary phenomena of scoliosis and the reaction of the spine to brace forces. 

To more accurately predict the outcome of brace treatment and the risk of scoliosis evolution would avoid the possibility of overbracing or a long period of conservative treatment in patients destined for surgery. This would prevent patients from unnecessary sacrifices and side effects, including psychological stress, repeated X-ray exposure and economic costs [10,11,12].

The outcomes of brace treatment are related to the interaction between mechanical and biological factors. The knowledge on the biomechanical factors that influence both the curve evolution and its treatment is limited. As a consequence, there is no definite evidence on the type of brace to use and its realization. An undefined number of braces is used, the empirical choice is more linked to the orthopedic surgeon’s habit than to evaluations about their efficacy and patient compliance. 

For this reason, in recent years, the International Society on Scoliosis Orthopedic and Rehabilitation Treatment (SOSORT) has pursued, with renewed interest, the study and evaluation of the different types of braces.

The biological factors that are identified in the literature as being involved in the progression of the curve and that influence the efficacy of brace treatment are multiple: sex, age, some genetic markers, Risser sign, curve pattern and rotation magnitude [13,14,15]. Other factors, specifically related to the treatment, are: prescribed hours of bracing, compliance and primary correction in brace [13,14,16].

Despite the high amount of dedicated research, it is still difficult to predict the outcome of treatment with a brace with sufficient accuracy. Therefore, convinced of the need to deepen our knowledge on prognostic factors, in recent years, we have assessed the importance of rotation [1,13]. The aim of these studies was to assess how the initial rotation affects the indications and the efficacy of the treatment, in particular, for the curves belonging to the “grey area”. The results showed that if the rotation is less than 20° Perdriolle and the Risser is between 0–2, the outcomes will be better [17]. This is because rotation values higher than 20° lead to hysteresis of the disc and the consequent inability to respond to the corrective actions of the brace, while a limited residual growth does not allow a sufficient correction to be obtained to ensure the stability of the scoliosis over the years [17]. Therefore, with the simultaneous evaluation of the Cobb angle, the vertebral rotation and the potential vertebral growth, it was possible to predict the outcome at the start of treatment. Moreover, the brace effectiveness is related to a longer daily time of correct use and to patient compliance with treatment schedules, as many papers have shown [18,19].

In another paper, we showed that wearing the brace for 22 h a day does not only block the process of evolution of AIS and juvenile idiopathic scoliosis (JIS) but also allows a significant recovery of the curve. Additionally, to remove the brace for more than 1 month a year has no effect on the treatment outcome. On the other hand, to exceed 2 months a year of bracing discontinuation and wearing the brace just overnight is associated with a greater risk of curve progression. The type of brace influences the compliance. Patients show higher adherence with Progressive Action Short Brace (PASB) use than a Lyon or Milwaukee brace. Furthermore, it is interesting that patients with AIS have a better compliance with brace treatment than those with JIS. Furthermore, PASB enables good correction both in adolescent and juvenile curves.

The degree of initial correction of the curve has been reported to predict the outcome: patients with an improved initial correction rate (ICR) could have a greater chance of success [20,21,22,23,24]. The limit value of the ICR for predicting the outcome remains controversial. Indeed, the literature shows correction values ranging from 10% to 60% [20,22,23].

The aim of this study was to evaluate if the initial correction rate could be predictive of the bracing outcome and to determine the role of some mechanical and biological parameters in ICR, specifically, Cobb degrees, Risser sign and Pedriolle degrees.

## 2. Materials and Methods

This is an observational controlled cohort study nested in a prospective clinical on-going database that includes 1536 patients treated for idiopathic scoliosis.

This study was conducted in accordance with the World Medical Association Declaration of Helsinki of 1975, as revised in 1983, and informed consent forms to permit clinical data use for research purposes were signed by all the participants. 

Among the 1536 patients, 446 patients who met the inclusion criteria were included. The inclusion criteria were: age > 10 years old, Risser 0–4 at the beginning of treatment, full-time brace prescription, no previous treatment, Curve magnitude (CM) 20–50° Cobb and radiographs at baseline (t1) after 3 months of treatment (t2) and after at least 3-year follow-up (t3). Curves between 20° and 25° Cobb degrees were included only if there was a documented progression. This condition was defined as an increase of more than 5° in CM (Cobb’s method) on two consecutive X-rays taken at 6-month interval. The minimum duration of follow-up was 24 months after the end of treatment. 

During bracing treatment (t2), X-rays were performed in-brace, at baseline (t1) and follow-up control (t3) radiographs were performed without brace. 

### 2.1. Bracing Protocol

The patients with thoracolumbar and lumbar curves were treated with PASB, instead of thoracic and double major curves with a Lyon or Milwaukee brace.

All patients had an indication of full-time bracing (20–22 h). 

The same physician followed all the patients from the treatment starting from the last follow-up, in order to improve treatment adherence. Since Risser was 3 or less, controls were performed every two months and every three months in Risser 4 and 5. Such close checks (two or three months) were useful to maximize bracing efficacy over time allowing brace adjustments due to curve or body shape if needed. Consent was obtained to supervise and strengthen patient compliance.

Compliance to treatment was verified during every clinical assessment by in-person interviews. Patients’ correct use of the brace was ratified by their parents and indirectly by the measurement of prominence. In the event that the prominence or curve worsened, patients’ behavior was deeply analyzed, and the physician incited a stronger parental involvement.

When ring-apophysis fusion was seen to begin, from a latero-lateral (LL) X-ray view, brace weaning started. This time corresponds to a Risser sign 4 or 5 from an antero-posterior (AP) standing X-rays view. Weaning was considered a 2 to 4 h bracing reduction every 2 months.

### 2.2. Statistical Analysis

In order to allow an easy comparison of the study results with other studies in the recent literature, three possible outcomes were distinguished, as recommended by the SRS committee: curve correction (CM t3–t1 ≤ −5° Cobb), curve stabilization (CM t3–t1 > −5° and < 5° Cobb) and curve progression (CM t3–t1 ≥ 5° Cobb).

The success of brace treatment at t3 (Success = (t1–t3) > 10 Cobb’s degree) was defined as a curve correction of >10 degrees. This cut-off, higher than the SRS committee direction, was chosen to allow a stronger statistical analysis. The success group and the failure group were compared in terms of the Risser sign, initial Cobb, initial Perdriolle and in-brace Initial Correction Rate. ICR was defined as ((Cobb t1–Cobb t2)/Cobb t1) × 100. 

To determine the independent predictors of the bracing outcome, a logistic regression model was created. We used Pearson’s correlation to test the ICR versus other variables.

Satistix 9.0 (Analytical Software, Tallahasse, FL, USA) was used for statistical analysis and calculations. 

## 3. Results

A definite outcome was recorded for 446 patients, 397 females (88.9%) and 49 males (11.1%), with a mean age of 12.67 ± 1.86 years at t1. The length of follow-up was 61.86 ± 54.12 (range 36–268) months. 

**Figure 1:**
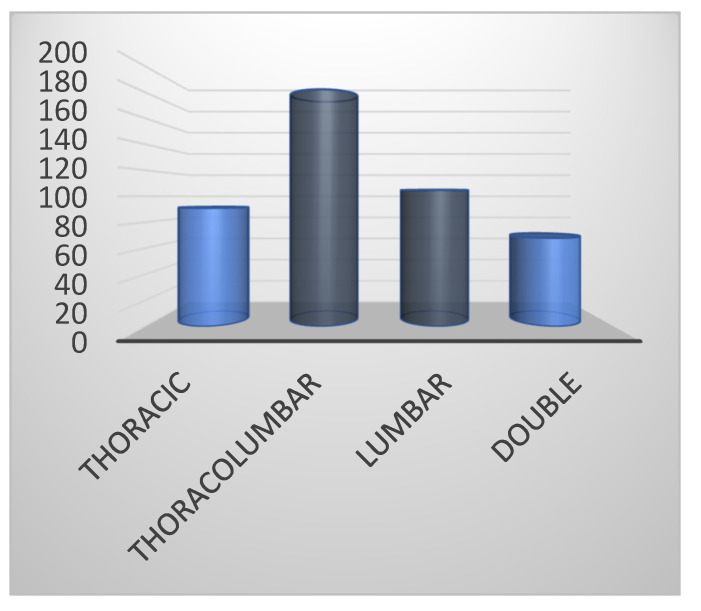
Curve type distribution.

Curve types were: thoracic (*n* = 91; 20.3%), thoracolumbar (*n* = 182; 40.8%), lumbar (*n* = 105; 23.6%) and double (*n* = 68; 15.3%) (Figure 1). 

The PASB was prescribed in 243 patients, and the Lyon or Milwaukee brace in 203 patients. 

**Figure 2:**
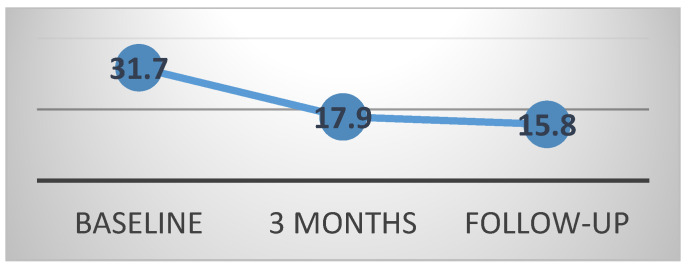
Changes in CM over time.

**Figure 3:**
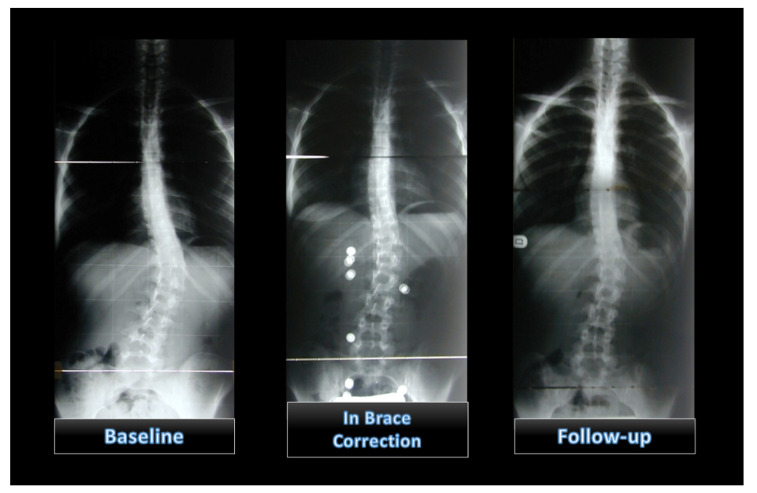
Patient X-rays that show changes over time.

Changes in the CM over time were statistically significant (*p* for trend <0.0001), with a mean value of 31.75 ± 8.8° Cobb at the start of treatment (t1), 17.91 ± 8.62 SD at 3 months (t2) and 15.87 ± 11.49° Cobb at follow-up (t3). Perdriolle at baseline was 13.20 ± 4.90 SD. (Figure 2 and Figure 3).

The Risser sign mean value was 1.84 (MAD 1, range 0–4); instead, the initial correction rate mean value was 45.1%, with a range of 6.9–100%. 

The ICR significantly correlates with the initial Perdriolle (*p* < 0.0001 and r = 0.3619).

Overall, 423 patients (94%) obtained a curve correction, while curve stabilization was achieved in 18 cases (5%). Five patients experienced curve progression (1%), and six patients were subsequently recommended for surgery because at follow-up the curve was over 45°. 

### Logistic Regression

For the logistic analysis, although the literature determines the success of a correction > 5° Cobb, we adopted a more strict threshold of success. Therefore, we established a double value threshold, identifying success only at a correction > 10° Cobb (Table 1). 

Instead, the Risser sign was dichotomized, assuming that from Risser 0 to Risser 2, there is a high growth potential (Risser < 3 = 1), and from Risser 3 to Risser 4, there is a low growth potential (Risser ≥ 3 = 0).

The logistic regression models with 0.5 cut-off provided a regression equation for logit:

L: L = −2.1907 + 1.8074 Risser + 0.0093 ICR (*p* = 0.998; Dev = 358). 

Constant *p* < 10^−4^. Coefficients: Risser *p* < 10^−4^, ICR *p* < 10^−4^. 

Factors that contribute the most to the positive result prediction are ICR and Risser. At the same time, the initial rotation significantly affected the final positive result, but the best prediction was obtained by considering ICR and Risser alone. The ICR and Risser coefficients reached a significance value of *p* < 10^−4^.

The decision-making matrix can be built (Table 2) and provides the diagnostic table (Table 3).

The method is highly predictive, with an accuracy of 84.74%, and shows a sensitivity of 94.5%. It appears particularly appropriate for positivity prediction (VP +: 87%).

The Odds Ratio underlined that the Risser sign is the most powerful predictor of the bracing outcome, with a value of 6.09 (95% CI = 3.62–10.26) compared with the ICR, 1.06 (95% CI = 1.04–1.08).

## 4. Discussion

In the literature, brace treatment effectiveness has been well documented by several studies. Despite the high amount of dedicated research, the role of the biological and mechanical factors is not completely clear; indeed, it is still difficult to predict the outcome of treatment with a brace with sufficient accuracy. 

In the study of Xu, the Risser sign was reported to be the independent predictor with an OR of 1.23, but its sensitivity was only 41.7% [16]. Another recent study reviewed a total of 488 AIS patients, and it was found that bracing outcome was significantly associated with the initial Risser sign, initial age and ICR. 

In this study, the ICR was the most powerful predictor of the bracing outcome, with an OR of 9.61, compared with an OR of 2.29 for the Risser sign [23]. However, these results disagree with those of our study in which the Risser sign was more significant, with a value of 6.09 compared with the ICR 1.06. 

In the current study, the relationship between ICR and the outcome was studied for a better understanding of brace treatment; although ICR has been discussed, no one has studied the role of biological and mechanical factors. However, in patients with idiopathic scoliosis, a central role in the evolution of deformities is played by the interaction between biological and mechanical factors. The anatomical changes in the scoliotic spine, change the system geometry, inducing a constraint reaction modification, thereby provoking a different stress load distribution model. In the deformed spine, the action of the loads translates as concentrations of tensions in specific vertebral areas, of the disks and of the capsulo-ligamentous apparatus. 

In the growing spine, these abnormally distributed forces can stimulate an asymmetrical growth of vertebral bodies and a neural arch. As a consequence, the scoliosis development during growth is the expression of a progressive deformation of the vertebrae that is part of the curve: “vicious cycle model”.

The braces, applying external constraints, modified the mechanical behavior of the scoliotic spine and its natural dynamics. Obviously, any corrective effect on biological structures can be stimulated only when a vertebral remodeling can be determined by mechanical actions. This can happen only if there is sufficient residual growth potential and if the visco-elastic structures are able to sufficiently react to the action imposed. Therefore, it is necessary for the inter-vertebral disks of the scoliotic curve to work within linear elasticity boundaries. When the mechanical action is efficient, it promotes vertebral remodeling and the recovery of a symmetric vertebral growth. These are fundamental requisites for healthy spinal growth and to obtain an improvement of the curve [3].

This allows understanding the role played by the different components in the prediction of the outcome. The initial correction and the residual growth are fundamental. Without initial correction, no new model of stress load distribution is produced. To obtain this, it is necessary that the initial degree of rotation is not so severe as to modify the visco-elastic characteristics of the intervertebral discs and that full time bracing is respected (high compliance).

It is demonstrated that a combination of the ICR and Risser sign could facilitate the accurate prediction of the bracing outcome. 

The limitations of the study are that it is retrospective; we cannot state why some patients did not complete their treatment (high worsening of curve, total recovery, etc.); and patient compliance was assessed through patient-reported surveys. Therefore, these variables could have affected the study outcomes.

## 5. Conclusions

This study confirms that immediate in-brace correction is essential to predict brace treatment outcomes. The most remarkable high chance of bracing success (87%) is for patients with a lower Risser sign and high correction rate in-brace.

## Figures and Tables

**Table 1:** Treatment efficacy.

Cobb T1–T3	Number of Cases	%	ES%
>5°	424	94.4	1.1
>10°	346	77.7	2
>15°	221	49.3	2.4
>20°	105	23.4	2

**Table 2:** Decision-making matrix (attended outcome: T+ or T−; real outcome: success or failure).

	Success	Failure
T+	327	49
T−	19	51
TOT	346	100

**Table 3:** Diagnostic table.

Sensitivity	94.5%
Specificity	51%
Predicting value (VP) +	87%
Predicting value (VP) −	73%
Accuracy	84.7%

## Data Availability

Datasets generated and/or analyzed during the current study are available from the corresponding author on reasonable request.
